# Erratum: Su, T.Y., et al. Effects of Heavy Metal Exposure on Shipyard Welders: A Cautionary Note for 8-Hydroxy-2′-Deoxyguanosine. *Int. J. Environ. Res. Public Health* 2019, *16*, 4813

**DOI:** 10.3390/ijerph18073557

**Published:** 2021-03-30

**Authors:** Ting-Yao Su, Chih-Hong Pan, Yuan-Ting Hsu, Ching-Huang Lai

**Affiliations:** 1 Graduate Institute of Life Sciences, National Defense Medical Center, Taipei 114, Taiwan; timothy80329@gmail.com (T.-Y.S.); misara116@gmail.com (Y.-T.H.); 2 Institute of Labor, Occupational Safety and Health, Ministry of Labor, New Taipei City 221, Taiwan; chpan@mail.ilosh.gov.tw; 3 School of Public Health, National Defense Medical Center, Taipei 114, Taiwan; 4 National Institute of Environmental Health Sciences, National Health Research Institutes, Miaoli 350, Taiwan

The authors wish to correct the following in this paper [1].

The fourth author, Ching-Huang Lai, should be affiliated with:

^1^  Graduate Institute of Life Sciences, National Defense Medical Center, Taipei 114, Taiwan
^3^  School of Public Health, National Defense Medical Center, Taipei 114, Taiwan

The authors would like to apologize for any inconvenience caused to the readers by this change.
